# Spatial Learning Drives Rapid Goal Representation in Hippocampal Ripples without Place Field Accumulation or Goal-Oriented Theta Sequences

**DOI:** 10.1523/JNEUROSCI.2479-21.2022

**Published:** 2022-05-11

**Authors:** Brad E. Pfeiffer

**Affiliations:** Neuroscience Graduate Program, Department of Neuroscience, Peter O'Donnell Jr. Brain Institute, University of Texas Southwestern Medical Center, Dallas, Texas 75390

**Keywords:** hippocampus, place cell, replay, sharp-wave/ripple, spatial memory, theta oscillation

## Abstract

The hippocampus is critical for rapid acquisition of many forms of memory, although the circuit-level mechanisms through which the hippocampus rapidly consolidates novel information are unknown. Here, the activity of large ensembles of hippocampal neurons in adult male Long-Evans rats was monitored across a period of rapid spatial learning to assess how the network changes during the initial phases of memory formation and retrieval. In contrast to several reports, the hippocampal network did not display enhanced representation of the goal location via accumulation of place fields or elevated firing rates at the goal. Rather, population activity rates increased globally as a function of experience. These alterations in activity were mirrored in the power of the theta oscillation and in the quality of theta sequences, without preferential encoding of paths to the learned goal location. In contrast, during brief “offline” pauses in movement, representation of a novel goal location emerged rapidly in ripples, preceding other changes in network activity. These data demonstrate that the hippocampal network can facilitate active navigation without enhanced goal representation during periods of active movement, and further indicate that goal representation in hippocampal ripples before movement onset supports subsequent navigation, possibly through activation of downstream cortical networks.

**SIGNIFICANCE STATEMENT** Understanding the mechanisms through which the networks of the brain rapidly assimilate information and use previously learned knowledge are fundamental areas of focus in neuroscience. In particular, the hippocampal circuit is a critical region for rapid formation and use of spatial memory. In this study, several circuit-level features of hippocampal function were quantified while rats performed a spatial navigation task requiring rapid memory formation and use. During periods of active navigation, a general increase in overall network activity is observed during memory acquisition, which plateaus during memory retrieval periods, without specific enhanced representation of the goal location. During pauses in navigation, rapid representation of the distant goal well emerges before either behavioral improvement or changes in online activity.

## Introduction

Rapid acquisition of many forms of sequential memory, including spatial information, critically relies on proper hippocampal function (Morris et al., 1982), but the exact circuit mechanisms through which the hippocampus contributes to the initial formation and use of spatial memory remain unclear. Positional information is encoded in the hippocampus via the spatially restricted firing patterns of individual hippocampal pyramidal neurons, termed place cells (O'Keefe and Dostrovsky, 1971; Moser et al., 2015). While the activity of place cells is ideal for encoding an animal's current location, successful navigation necessarily requires representation of a nonlocal destination and calculation of a plausible path to reach that location. Although several studies have observed that learned goal locations display altered place cell representation (Hollup et al., 2001; Lee et al., 2006; Hok et al., 2007; Dupret et al., 2010; Mamad et al., 2017; Kaufman et al., 2020), it is not evident how changes in ensemble activity exclusively at a goal location are sufficient to facilitate goal-directed navigation from a distant starting point. During periods of both movement and immobility, the hippocampus expresses distinct internally generated sequences which reflect virtual paths through the current environment (Wilson and McNaughton, 1994; Skaggs et al., 1996; Foster and Wilson, 2006, 2007; Diba and Buzsáki, 2007; Johnson and Redish, 2007; Karlsson and Frank, 2009; Gupta et al., 2010; Singer et al., 2013; Wikenheiser and Redish, 2015). Because internally generated sequences encode nonlocal spatial information, they have been postulated as a network mechanism which may underlie navigation to distant goals (Carr et al., 2011; Foster and Knierim, 2012; Redish, 2016).

During active navigation, the hippocampal network is organized by the ongoing 6-12 Hz theta oscillation (Skaggs et al., 1996). Within each theta wave, sequences of place cells are temporally ordered to encode brief sweeps of spatial information, starting at the rat's current location and extending a short distance (Foster and Wilson, 2007; Johnson and Redish, 2007; Gupta et al., 2012; Zheng et al., 2016). Recent work indicates that theta sequences encode prospective information about future behavior (Kay et al., 2020; Wang et al., 2020) and can represent distant goal locations (Wikenheiser and Redish, 2015), suggesting that they may facilitate navigational decision-making. While theta sequences do not appear to correlate to behavioral choice in a maze with two path options distant from a reward location (Kay et al., 2020), the content in theta sequences could theoretically be used to evaluate possible future outcomes, particularly as an animal approaches a goal. Indeed, given the relatively short spatial distances typically observed in theta sequences (Kay et al., 2020; Wang et al., 2020), biases in theta sequence representation toward goal locations may only arise as the animal draws nearer to a destination.

During periods of immobility, the hippocampus can generate temporally compressed spatial sequences encoded within sharp-wave/ripples (Buzsáki, 2015), termed replay (Foster and Wilson, 2006; Diba and Buzsáki, 2007). The content of replay can be diverse (Pfeiffer, 2020) but is typically correlated to an animal's recent past or immediately future behaviors (Diba and Buzsáki, 2007; Davidson et al., 2009; Singer et al., 2013; Ólafsdóttir et al., 2017; Xu et al., 2019; Gillespie et al., 2021). Several studies have reported that replay trajectories are biased to represent paths from a rat's current location to a recently learned location of either positive or negative salience (Pfeiffer and Foster, 2013; Ólafsdóttir et al., 2017; Wu et al., 2017; Carey et al., 2019; Xu et al., 2019), implicating replay in a decision-making process that evaluates future behavioral options. In addition, the paths encoded by replay are more tightly aligned with future behaviors than with past behaviors during a goal-directed navigational task (Pfeiffer and Foster, 2013; Singer et al., 2013; Ólafsdóttir et al., 2017; Xu et al., 2019). Consistent with a role for hippocampal replay in guiding navigation, cortical networks implicated in decision-making are coordinated with the hippocampus during replay (Peyrache et al., 2009; Jadhav et al., 2016; Shin et al., 2019) and interfering with replay during a navigational task impairs working memory performance (Jadhav et al., 2012). Importantly, recent studies have observed that, in some behavioral tasks, replay events more strongly encode paths to a previous goal location rather than a current goal location (Carey et al., 2019; Gillespie et al., 2021), suggesting that replay events are more compatible with retrieval or consolidation of prior experience rather than planning or prospective evaluation of future experience.

Thus, while sequentially organized activity during both theta sequences and ripple-based replay have been associated with the formation and/or retrieval of spatial memory, their precise contribution to goal-directed navigation remains unclear. In this study, large ensembles of hippocampal neurons were examined while rats performed a task requiring rapid acquisition and use of a novel spatial memory to quantify how the hippocampal network changes to facilitate this process.

## Materials and Methods

The dataset used in this study has been previously analyzed (Pfeiffer and Foster, 2013).

### Behavior and electrophysiological recording

All animal procedures were approved by the Johns Hopkins University Animal Care and Use Committee and followed National Institutes of Health animal use guidelines. A detailed description of the behavior and training has been described previously (Pfeiffer and Foster, 2013) and is summarized here. Four male adult Long-Evans rats were used in this study. Behavior during recording was performed in a 2 m × 2 m open arena with 30-cm-high walls and 36 identical, evenly spaced, 1.5-cm-diameter, 3-mm-deep conical reward delivery wells embedded into the floor such that the rim of each well was level with the floor. Each well could be independently filled without audible or visible cue. When filled, each well held ∼300 µl of chocolate milk. Recordings took place during the rats' light cycle. Behavioral training took ∼20-30 d. On each day of training, a different well was used as the Goal well such that no well was ever repeated as the Goal well. During recording, the rat was placed into one of the four corners of the arena and the Goal well was the only filled well. Once the rat found and consumed the Goal well, one of the remaining 35 Random wells was filled. When the rat found and consumed the filled Random well, the Goal well was filled. When the rat found and consumed the Goal well, another Random well was filled. This pattern was repeated until the end of the recording. No Random well was used twice for at least the first 19 trials, and no Random well was used more than once within 15 consecutive trials. Goal-seeking periods were defined as periods when the Goal well was filled; Random-foraging periods were defined as periods when a Random well was filled. Well boundaries were defined as borders equidistant from adjacent wells. Well entries were quantified as crossings of well boundaries (moving from one well to another).

A detailed description of the electrophysiological recording has been described previously (Pfeiffer and Foster, 2013) and is summarized here. Adult male rats were implanted with a microdrive array (25-30 g) containing 40 independently adjustable, gold-plated tetrodes aimed at area CA1 of dorsal hippocampus (20 tetrodes in each hemisphere; 4.00 mm posterior and 2.85 mm lateral to bregma). Postexperimental lesions confirmed that the recording locations were in area CA1 (Pfeiffer and Foster, 2013). Tetrode advancement took place over the course of 7-10 d, during which the rats continued to be trained in the task. Each tetrode consisted of a bundle of four 17.8 µm platinum/10% iridium wires (California Fine Wire), and each wire was electroplated with gold to an impedance of <150 mΩ before surgery. A bone screw near λ served as ground. All electrophysiological data were acquired at 32,556 Hz using a Neuralynx data acquisition system and an overhead video system recorded behavior at 60 Hz. Action potentials were detected as threshold (50 µV) crossings of 600-6000 Hz bandpass filtered data. Continuous local field potential (LFP) data were digitally filtered between 0.1 and 500 Hz and recorded at 3255.6 Hz.

Action potentials were assigned to individual units via manual clustering based on spike waveforms. A detailed description of cluster procedures and unit isolation quality from this study has been reported previously (Pfeiffer and Foster, 2013).

### LFP analysis

For each tetrode that recorded at least one place cell, a representative electrode was selected and the LFP signal was analyzed. To identify ripples, the LFP was bandpass filtered between 150 and 250 Hz, and the absolute value of the Hilbert transform of this filtered signal was smoothed (Gaussian kernel, SD 12.5 ms). This processed signal was averaged across all tetrodes, and ripples were identified as local peaks with an amplitude >3 SD above the mean, using only periods when the rat's velocity was <5 cm/s. The start and end boundaries for each ripple were defined as the point when the signal crossed the mean. To quantify theta in each session, a single centrally located electrode was identified in the pyramidal layer based on the observance of multiple single excitatory units and large amplitude ripple events with minimal sharp-wave deflection from that electrode. The raw LFP of this electrode was bandpass filtered between 6 and 12 Hz. Theta power was defined as the smoothed (Gaussian kernel, SD 300 ms) absolute value of the Hilbert transform, *z*-scored across the entire session. Only periods of active movement (velocity > 10 cm/s) were used to analyze theta.

### Place cell analysis and spatial decoding

Position was binned (2 cm) and position tuning curves (place fields) were calculated as the smoothed (2D Gaussian kernel, SD 4 cm) histogram of firing activity normalized by the time spent per bin. Only periods of time when the rat was moving faster than 5 cm/s were used to determine place fields. Units were considered to have a place field if the unit was classified as excitatory and the peak of the tuning curve was >1 Hz. Place fields for each unit were defined as contiguous spatial bins with >20% of the maximal firing rate (minimum of 20 bins). Spatial correlations were calculated as the linear correlation of smoothed firing rate across all spatial bins in which either cell had a non-zero smoothed firing rate. For in-field firing rate calculations, only the place field with maximal firing rate was considered. “Goal cells” were place cells with maximal firing nearer the Goal well than any other well. “Goal-adjacent cells” were place cells with maximal firing nearer any of the eight Random wells adjacent to the Goal well than any other well. Any other place cell was defined as a “Goal-distant cell.” Spatial information was calculated as previously described (Skaggs et al., 1993).

Spatial decoding was performed as previously described (Davidson et al., 2009; Pfeiffer and Foster, 2013). To quantify mean decoding error during movement, nonoverlapping time windows of 250 ms were used. Decoding error is defined as the distance between the rat's current location and the weighted mean of the posterior probability for each window.

### Replay analysis

For each ripple, the encoded position was estimated using a time window of 20 ms advanced in 5 ms steps. A sequence of locations (weighted mean of each decoding window) was calculated, and each ripple was then analyzed to identify events that met criteria to be classified as trajectory-encoding events. A trajectory-encoding replay required at least 15 consecutive decoding frames in which the decoded location moved <30 cm between frames but in which the start-to-end distance traveled across all decoded frames was >40 cm. These criteria eliminated ripples which either encoded a single, unmoving location (Denovellis et al., 2021) or which “jumped” randomly around the arena. For ripples that met the criteria, statistical significance was quantified via a Monte-Carlo *p* value using two shuffle methods: randomly shuffling cell identify and randomly rotating each cell's place field in both the *x* and *y* dimensions. The *p* value was calculated as (*n* + 1)/(*r* + 1), where *n* is the number of shuffles that met the criteria and *r* is the total number of shuffles. Events that encoded a trajectory with a *p* value <0.05 for both shuffle methods were classified as replays. For each replay event, representation of the well nearest the rat's current location was eliminated from future analysis, including all spatial bins closer to the current well than any other well. Thus, replay analysis focused exclusively on nonlocal representations. A ripple was considered to encode the Goal location if the sum of posterior probabilities across all decoding frames within a 30 cm × 30 cm box around the Goal well was >0.075.

### Theta sequence analysis

The trough of the theta-filtered oscillation was defined as 0°. The beginning (and end) of each theta oscillation was assigned to 60°. Only theta oscillations when the rat was moving >10 cm/s were included in theta oscillation decoding. To quantify decoding accuracy across each theta oscillation (see Fig. 5), each theta oscillation was decoded in a single window (spanning from 60° to 60°). For theta sequence decoding, the encoded position was estimated using a phase window of 60° (equivalent to 15-25 ms) advanced in 15° (equivalent to 3.5-7 ms) bins. The forward-encoding portion of each theta oscillation was defined as a period from 240° to 60° (Wang et al., 2020). All decoding windows for a given theta oscillation were averaged, and the resulting two-dimensional matrix was centered to the rat's current location and rotated by either the rat's current movement direction or the direction from the rat to the Goal well. The resulting two-dimensional matrix was then summed across the *y* axis to produce a relative representation normalized to the rat. Slope was calculated by quantifying a weighted best-fit line in the forward-encoding portion. The quadrant score was calculated as previously described (Feng et al., 2015). For subsampling control analyses, for each session, the mean number of action potentials in each theta oscillation was quantified during the Learning period. For each theta oscillation in the Retrieval period, action potentials were randomly removed to match this average. This subsampling was performed 1000 times for each theta oscillation during the Retrieval period. Slope and quadrant scores were calculated for each subsampled dataset, and the results were averaged for each theta oscillation.

### Statistics

Statistical analyses were performed via custom MATLAB code. Before ANOVA, Lilliefors test was used to assess normality (α = 0.05). All ANOVA *post hoc* multiple comparison tests used Tukey's honestly significant difference procedure. For two-sample comparisons, the Wilcoxon rank sum test was used. Detailed statistical information for each figure is provided in each figure legend.

### Figure color code

To provide visual structure to the data presentation, the following color code is used throughout all figures, other than Figures 2*D* and 8. Green represents analysis of random foraging periods; blue represents analysis of inhibitory neurons; red represents analysis of goal-adjacent excitatory neurons; cyan represents analysis of goal-distant excitatory neurons; and magenta represents analysis of goal-seeking periods.

### Data and code accessibility

All data and analysis code are available on request.

## Results

**Figure 1.**
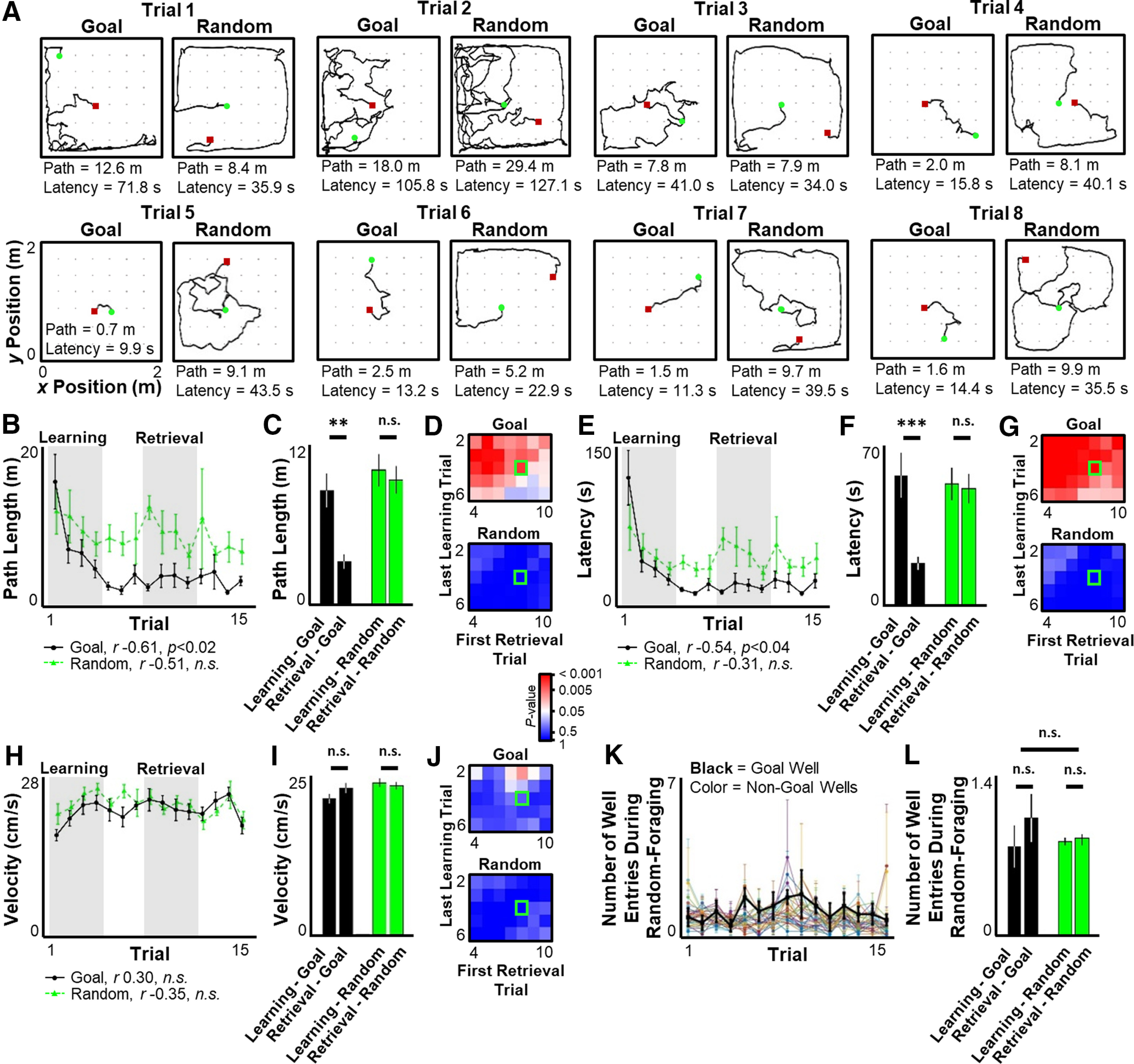
Improvement in behavioral performance across trials. ***A***, Quantified behavioral trajectories (black line) across the first 8 trials for one session during goal-seeking (Goal) and random-foraging (Random) components, with start (green circle) and end (red square) denoted. ***B***, ***E***, ***H***, For the first 15 trials across all sessions, mean ± SEM path length (***B***), latency (***E***), and mean velocity during active movement periods (***H***) during goal-seeking versus random-foraging segments. Pearson's linear correlation statistics for first 15 trials shown below graph. ***C***, ***F***, ***I***, Mean ± SEM of path length (***C***), latency (***F***), and velocity (***I***) during Learning versus Retrieval periods defined by shaded regions in ***B***, ***E***, and ***H***, respectively. ***D***, ***G***, ***J***, Significance matrix calculated as in ***C***, ***F***, and ***I***, but for different definitions of the Learning and Retrieval periods. Plotted is the *p* value for Learning versus Retrieval period. Highlighted square in ***D***, ***G***, and ***J*** represents the Learning and Retrieval period windows quantified in ***C***, ***F***, and ***I***. ***K***, For the first 15 trials, mean ± SEM number of times the rat entered a well partition during the random-foraging segment of the task for the Goal well (thick black line) and each other well (thin colored lines). ***L***, Mean ± SEM of Goal well (black) and average non-Goal well (green) crossings during random-foraging portion of Learning versus Retrieval periods. Statistical tests and results: ***C***, Two-way ANOVA. *n* = 8 sessions × 4 time points per group. Main effects: Learning versus Retrieval, *F*_(1,124)_ = 8.163, *p* = 0.005; Goal-seeking versus Random-foraging, *F*_(1,124)_ = 13.02, *p* = 0.0004; Interaction, *F*_(1,124)_ = 4.671, *p* = 0.0326. *Post hoc* multiple comparisons (Tukey's HSD): Learning-Goal versus Retrieval-Goal, adjusted *p* = 0.003; Retrieval-Goal versus Learning-Random, adjusted *p* < 0.0001; Retrieval-Goal versus Retrieval-Random, adjusted *p* = 0.0005; all other comparisons, adjusted *p* > 0.7. ***F***, Two-way ANOVA. *n* = 8 sessions × 4 time points per group. Main effects: Learning versus Retrieval, *F*_(1,124)_ = 8.332, *p* = 0.0046; Goal-seeking versus Random-foraging, *F*_(1,124)_ = 4.231, *p* = 0.0418; Interaction, *F*_(1,124)_ = 6.684, *p* = 0.0109. *Post hoc* multiple comparisons (Tukey's HSD): Learning-Goal versus Retrieval-Goal, adjusted *p* = 0.001; Retrieval-Goal versus Learning-Random, adjusted *p* = 0.0036; Retrieval-Goal versus Retrieval-Random, adjusted *p* = 0.0072; all other comparisons, adjusted *p* > 0.9. ***I***, Two-way ANOVA. *n* = 8 sessions × 4 time points per group. Main effects: Learning versus Retrieval, *F*_(1,124)_ = 0.7076, *p* = 0.4019; Goal-seeking versus Random-foraging, *F*_(1,124)_ = 4.086, *p* = 0.0454; Interaction, *F*_(1,124)_ = 2.213, *p* = 0.1394. *Post hoc* multiple comparisons (Tukey's HSD): all comparisons, adjusted *p* > 0.067. ***L***, Two-way ANOVA. *n* = 8 sessions × 4 time points × 1 well for Goal groups, *n* = 8 sessions × 4 time points × 35 wells for Random groups. Main effects: Learning versus Retrieval, *F*_(1, 2,300)_ = 0.7216, *p* = 0.3957; Goal versus Random crossings, *F*_(1, 2,300)_ = 0.1569, *p* = 0.6921; Interaction, *F*_(1, 2,300)_ = 0.4626, *p* = 0.4965. **p ≤ 0.01. ***p ≤ 0.001. n.s., *p* > 0.05.

Rats were trained to perform a spatial navigation task in a familiar open arena (2 m × 2 m) with 36 evenly spaced wells embedded in the arena floor arranged in a 6 × 6 grid (Fig. 1*A*). The task required alternation between random foraging to find reward in an unpredictable location (Random wells) and goal-directed navigation to a recently learned, predictable reward location (the Goal well). The location of the Goal well remained in the same location across all trials of a given day but changed unpredictably to new locations across days, forcing the rats to learn the new Goal location each day. Once a location was used as a Goal, it was never reused as a Goal for any subsequent day, forcing the rats to establish a new memory and use novel navigational strategies every day. After rats reached criterion performance on the task, they were implanted with large tetrode arrays to monitor neural activity during performance across multiple days. Eight sessions were recorded (4 rats; two sessions per rat), with between 80 and 263 simultaneously recorded CA1 hippocampal units in each session. The analyses in this study sought to identify experience-dependent changes in hippocampal circuit function during spatial learning.

Behavioral performance was quantified to identify periods of initial memory acquisition and subsequent memory use. Analysis was initially restricted to the first 15 trials of each session to avoid behavioral changes in later trials caused by satiety. In each session, rats showed significant improvement in their ability to navigate to the new Goal well location, but not to the Random well (Fig. 1*B*,*E*). Behavioral performance curves for goal-directed navigation reached asymptote on Trial 6, at which point neither path length nor latency improved further (Fig. 1*B*,*E*, Trials 1-6, path length, *r* = −0.905, *p* < 0.02, latency, *r* = −0.841, *p* < 0.04; Trials 7-15, path length, *r* = −0.231, *p* > 0.5, latency, *r* = 0.144, *p* > 0.7). Based on these observations, Trials 1-4 were conservatively defined as the Learning phase, when goal-directed navigation consistently improved, and Trials 8-11 as the Retrieval phase, when peak performance was sustained. The Learning phase was hypothesized to be a period when the location of the Goal well was being incorporated into memory, and the Retrieval phase was hypothesized to be a period when the memory of the Goal location was recalled to more efficiently guide behavior. Navigation to the Goal well significantly improved from the Learning to Retrieval phase, but navigation to unpredictable, Random wells did not change during this period (Fig. 1*C*,*F*). To ensure that the choice of Learning and Retrieval windows did not bias the observation of behavioral improvement, a wide range of Learning and Retrieval windows were tested (Fig. 1*D*,*G*). Improved performance in the goal-seeking portion of the task was largely independent of the specific windows used to define the Learning or Retrieval phases (Fig. 1*D*,*G*), although when the Learning phase was extended to later trials with short latencies and path lengths, the difference between Learning and Retrieval was predictably diminished. Overall running speed did not change across the task and thus did not account for behavioral improvement (Fig. 1*H–J*).

To ensure proper interpretation of these data, it is important to assess whether rats understood the underlying rules of the task (consistent alternation between goal-directed navigation and random foraging) rather than learning a more basic, but false rule that the Goal well is simply more likely to be rewarding than other locations. If a rat learned the false rule, it would be expected to repeatedly return to the Goal well during the random-foraging segment of the task. However, during the random-foraging component, rats did not cross the Goal well more often than any other non-Goal well, even during the Retrieval phase when their behavior during the goal-seeking segment was strongly biased toward the Goal well (Fig. 1*K*,*L*). Thus, rats behaved differently during the two segments of the task and often did not return to the Goal well until after finding a baited Random well, displaying a basic understanding of the overall rules of the task. Specific comparison between the Goal-seeking and Random-foraging components of the task could therefore be performed to assess how the hippocampal circuit may facilitate storage and retrieval of the goal location.

**Figure 2.**
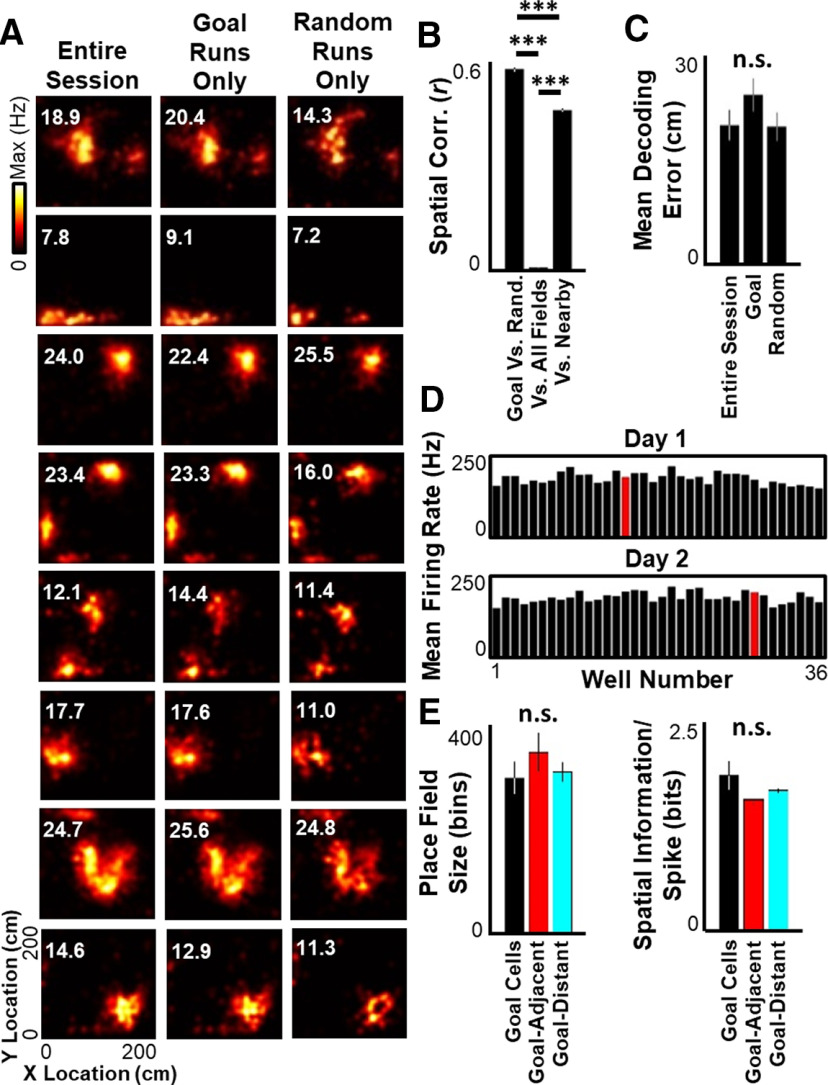
Spatial representation does not change during goal-seeking versus random-foraging. ***A***, Spatial tuning maps (place fields) for 8 example neurons calculated using spikes and position information either during the entire session (left), restricted to the goal-directed segments of the task (middle), or restricted to the random-foraging segments of the task (right). Maximum firing rate listed for each tuning curve in top left. ***B***, Left, Mean ± SEM spatial correlation between place fields for each cell calculated during only goal-directed segments and place fields calculated during only random-foraging segments. Middle, Correlation between entire-session place fields for all recorded cells. Right, Correlation between place fields of different cells with overlapping fields (spatial bin of maximal firing <10 cm apart). ***C***, Mean ± SEM decoding error across the entire session (using nonoverlapping decoding windows of 250 ms) when decoding with place fields calculated during the entire session (left), during only goal-directed segments (middle), or during only random-foraging segments (right). ***D***, Mean excitatory population firing rate at each well (Goal well in red) for all 4 rats on experimental days 1 (top) and 2 (bottom). ***E***, Mean ± SEM place field size (left) and information per spike (right) for place cells with peak firing closer to the Goal well than any other well (“Goal cells”), place cells with peak firing nearest the eight wells adjacent to the Goal well (“Goal-Adjacent”), and place cells with peak firing elsewhere in the arena (“Goal-Distant”). Statistical tests and results: ***B***, One-way ANOVA. *n* = 1172 field pairs for Goal/Random; 101,482 field pairs for All-Fields; 1135 field pairs for Nearby. Main effects: *F*_(2, 103,786)_ = 12,130.39, *p* < 10^−6^; *post hoc* multiple comparisons (Tukey's HSD): all comparisons adjusted *p* < 10^−8^. ***C***, One-way ANOVA. *n* = 8 sessions per group. Main effects: *F*_(2,21)_ = 1.3429; *p* = 0.2826. ***E***, Left, One-way ANOVA. *n* = 44 Goal cells, 235 Goal-adjacent cells, 909 Goal-distant cells. Main effect: *F*_(2,1185)_ = 0.4388; *p* = 0.6449. ***E***, Right, One-way ANOVA. *n* = 44 Goal cells, 235 Goal-adjacent cells, 909 Goal-distant cells. Main effect: *F*_(2,1185)_ = 2.2784; *p* = 0.1029. ***p ≤ 0.001. n.s., *p* > 0.05.

Given the distinct behaviors and mnemonic requirements for goal-directed navigation versus random foraging in this task, place cell representation was independently quantified in the two behavioral components to determine whether the hippocampal network differentiated between these two behavioral tasks in the same arena. For each cell, place field firing maps were generated using neural activity and position information restricted to either goal-seeking or random-foraging segments; 1172 of 1188 (98.7%) putative excitatory neurons had place fields in both behavioral conditions, and place fields were strikingly similar across behavior categories (Fig. 2*A*). Spatial correlations between goal-seeking versus random-foraging place fields from the same cell were significantly higher than between place fields from different cells with similar peak firing locations (Fig. 2*B*). In addition, there was no difference in the accuracy of population-level spatial representations when decoding with place fields calculated either during the entire session, during only goal-seeking periods, or during only random-foraging components of the task (Fig. 2*C*). Thus, gross differences were not observed in spatial coding between the two components of the task, arguing against global place field remapping (Colgin et al., 2008) as a contributing factor for goal-directed navigation.

Prior studies have reported increased place cell activity at learned goals (Hollup et al., 2001; Hok et al., 2007; Gauthier and Tank, 2018; Sato et al., 2020). Enhanced representation of rewarding locations across the hippocampal network has been postulated as a mechanism that may influence goal-directed navigation. To determine whether the Goal location was more strongly encoded by the hippocampal ensemble, the arena was divided into 36 partitions centered on each well and the population firing rate in each partition was quantified during periods of active movement. All wells, including the Goal well, had similar levels of total neural activity (Fig. 2*D*, Goal = 196.1 ± 36.9 Hz across all recorded cells; non-Goal = 185.4 ± 5.7 Hz; Wilcoxon rank sum *p* > 0.7). In addition, cells with maximal activity at the Goal well (“Goal cells”) did not have significantly different place field sizes or spatial information per spike than cells with maximal activity at wells adjacent to (“Goal-Adjacent cells”) or distant from (“Goal-Distant cells”) the Goal well (Fig. 2*E*). Therefore, the recently learned Goal location had similar spatial representation quality as any other location in the arena, and changes in goal-specific encoding across the hippocampal place cell network are unlikely to explain successful goal-directed navigation in this behavioral task.

Thus, no obvious bias in Goal representation was observed when activity across the entire session was combined, arguing against a global or preexisting mechanism for hippocampal goal-directed navigation in the current task. Having defined periods of both rapid memory acquisition and post-learning memory use, changes in the circuit-level hippocampal function were next quantified across the Learning and Retrieval phases to identify network features which may account for behavioral improvement.

**Figure 3.**
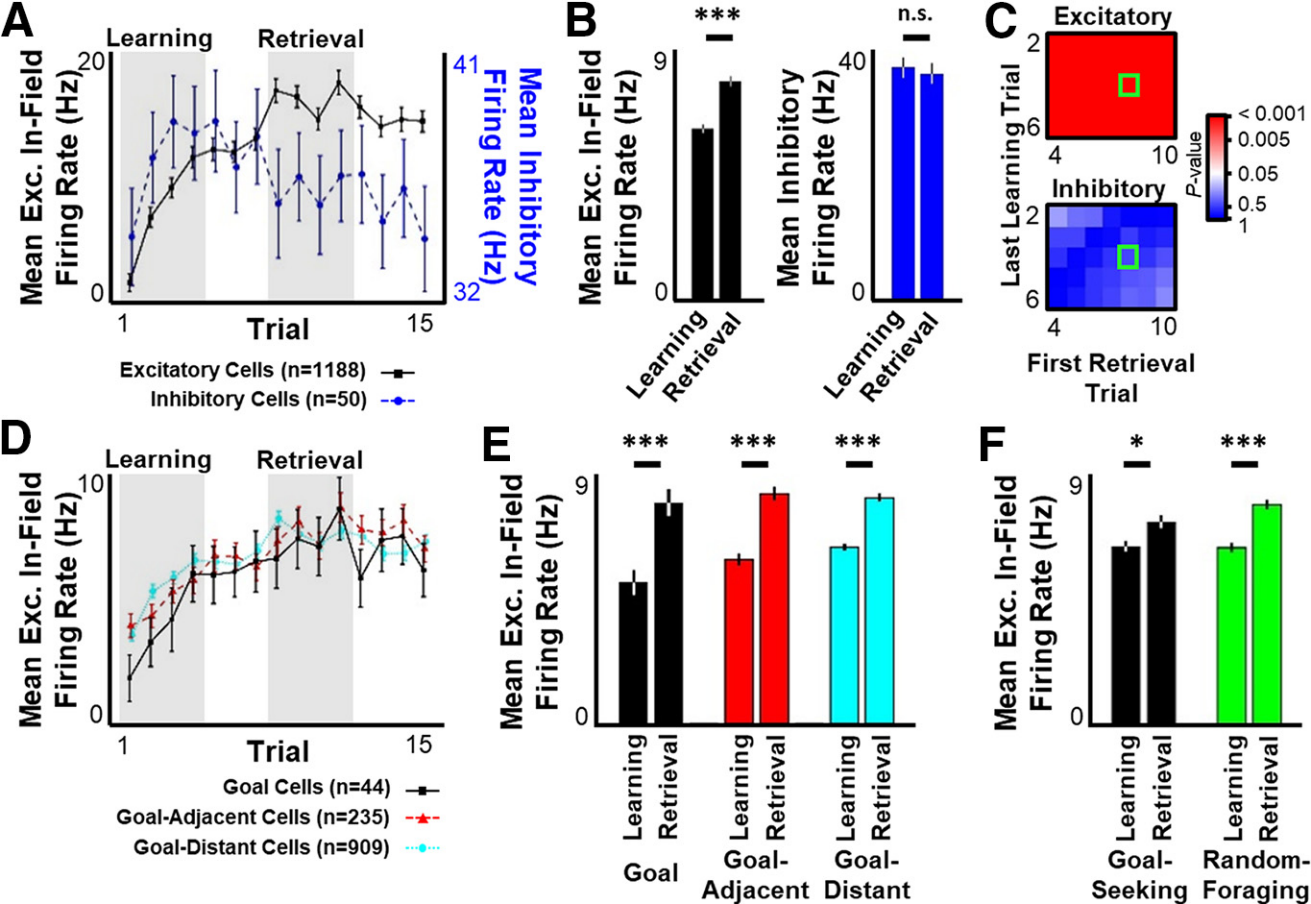
Excitatory population activity increases across learning. ***A***, For the first 15 trials across all sessions, mean ± SEM in-field firing rate per putative excitatory cell (black) or overall firing rate per putative inhibitory cell (blue). ***B***, Mean ± SEM excitatory in-field (left) or inhibitory (right) firing rate during Learning and Retrieval periods defined by shaded regions in ***A***. ***C***, Significance matrix as in Figure 1*D* for excitatory in-field (top) or inhibitory (bottom) firing rate. ***D***, Same as in ***A***, separated by Goal cells (black), Goal-adjacent cells (red), and Goal-distant cells (cyan). ***E***, Mean ± SEM in-field firing rate for Goal cells (left), Goal-adjacent cells (middle), and Goal-distant cells (right) during Learning and Retrieval periods defined by shaded regions in ***A***. ***F***, Mean ± SEM excitatory in-field firing during goal-seeking (black) or random-foraging (green) segments during Learning and Retrieval periods defined by shaded regions in ***A***. Statistical tests and results. ***B***, Left, Wilcoxon rank sum test. *n* = 1188 cells. *p* < 10^−10^. ***B***, Right, Wilcoxon rank sum test. *n* = 50 cells. *p* = 0.4380. ***E***, Two-way ANOVA. *n* = 44 Goal cells, 235 Goal-adjacent cells, 909 Goal-distant cells × 4 time points × 8 sessions. Main effects: Learning versus Retrieval, *F*_(2, 6970)_ = 76.53, *p* < 0.0001; Cell type, *F*_(2, 6970)_ = 2.052, *p* = 0.1286; Interaction, *F*_(2, 6970)_ = 2.054, *p* = 0.1284. *Post hoc* multiple comparisons (Tukey's HSD): all Learning versus Retrieval for same cell type, adjusted *p* < 0.0007; all across-cell-type for Learning, adjusted *p* > 0.14; all across-cell-type for Retrieval, adjusted *p* > 0.9. ***F***, Two-way ANOVA. *n* = 1188 cells. Main effects: Learning versus Retrieval, *F*_(1, 4187)_ = 40.07, *p* < 0.0001; Goal-seeking versus Random-foraging, *F*_(1, 4187)_ = 2.145, *p* = 0.1431; Interaction, *F*_(1, 4187)_ = 3.056, *p* = 0.0805. *Post hoc* multiple comparisons (Tukey's HSD): all Learning-Goal versus Retrieval-Goal, adjusted *p* = 0.0109; Learning-Random versus Retrieval-Random, adjusted *p* < 0.0001; Learning-Goal versus Retrieval-Random, adjusted *p* < 0.0001; Learning-Random versus Retrieval-Goal, adjusted *p* = 0.0047; all other comparisons, adjusted *p* > 0.12. *p ≤ 0.05. ***p ≤ 0.001. n.s., *p* > 0.05.

To assess whether overall neural activity changed as a function of learning, population firing rates were quantified on a per-trial basis. Average in-field firing rates for putative excitatory neurons consistently increased throughout the Learning phase, in agreement with prior work (Michon et al., 2021), and plateaued during the Retrieval phase (Fig. 3*A–C*). The per-trial in-field firing rate of excitatory neurons was highly correlated with behavioral performance to find the Goal, but not Random well (firing rate vs Goal latency, *r* = −0.151, *p* < 0.0175; vs Goal path length, *r* = −0.160, *p* < 0.0120; vs Random latency, *r* = −0.0096, *p* > 0.88; vs Random path length, *r* = 0.024, *p* > 0.71). Importantly, there was no difference between Goal cells, Goal-Adjacent cells, or Goal-Distant cells in experience-dependent firing rate changes (Fig. 3*D*,*E*), indicating that the increased activity of excitatory neurons was not specific to representation of the Goal well, but instead reflected overall increased activity across the hippocampal network. Furthermore, excitatory in-field firing rates increased during both goal-seeking and random-foraging segments of the task (Fig. 3*F*). Thus, increased hippocampal activity was not limited only to periods when memory retrieval was necessary to perform the task. Unlike excitatory neurons, putative inhibitory neurons showed no sustained or significant change in firing rate across the experiment, despite a brief increase in activity over the first few trials (Fig. 3*A–C*), indicating cell-type specificity in how learning influenced hippocampal activity.

**Figure 4.**
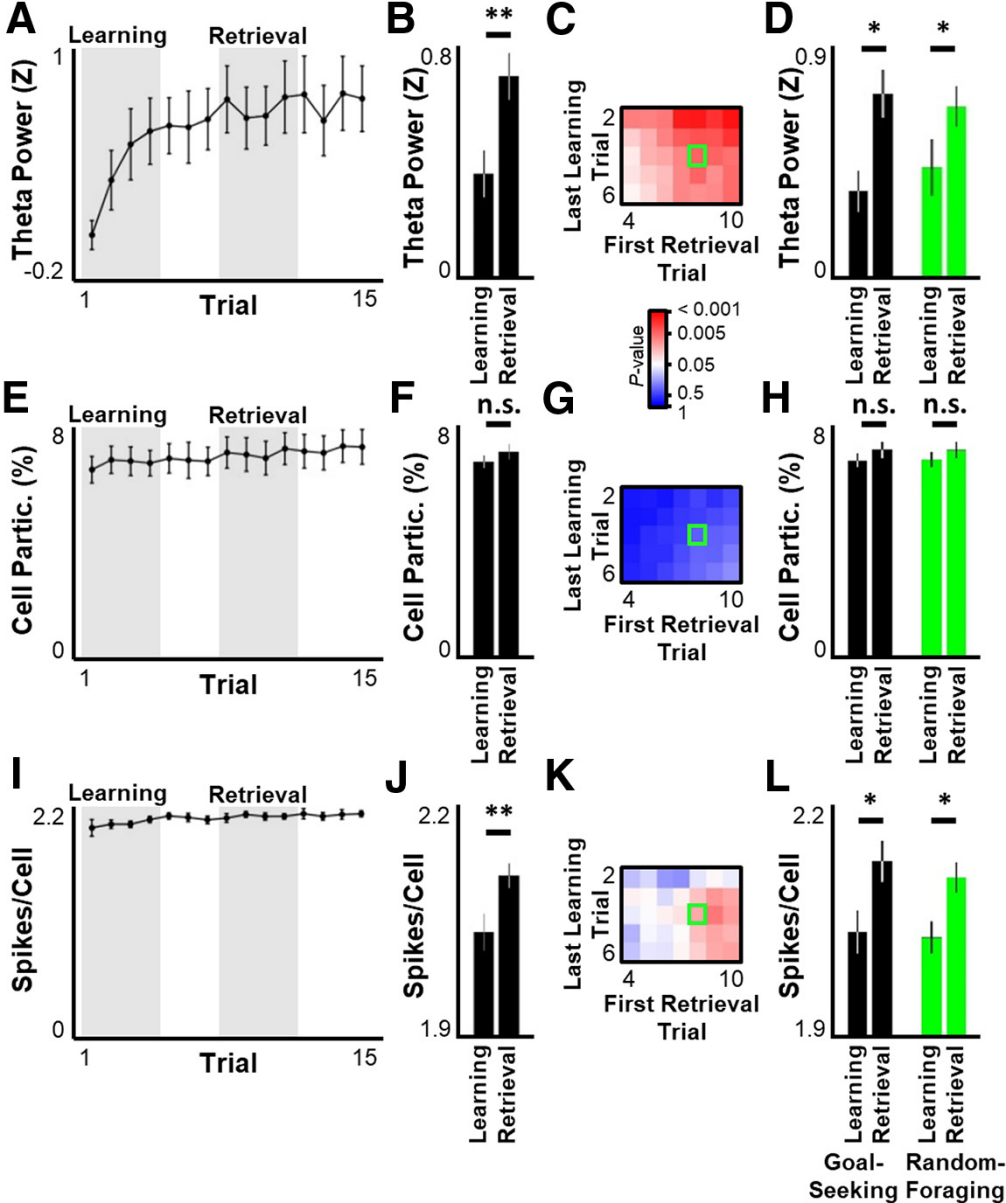
Theta power increases across learning. ***A***, ***E***, ***I***, For the first 15 trials across all sessions, mean ± SEM theta power (***A***), percent of recorded cells active per theta oscillation (***E***), and number of spikes per participating cell per theta oscillation (***I***). ***B***, ***F***, ***J***, Mean ± SEM of theta power (***B***), cell participation (***F***), and spikes per participating cell (***J***) during Learning versus Retrieval periods. ***C***, ***G***, ***K***, Significance matrix as in Figure 1*D* for theta power (***C***), cell participation (***G***), and spikes per participating cell (***K***). ***D***, ***H***, ***L***, Same as in ***B***, ***F***, and ***J***, separated into goal-seeking (black) and random-foraging (green) segments. Statistical tests and results: ***B***, Wilcoxon rank sum test. *n* = 8 sessions × 4 time points per group. *p* = 0.003975. ***D***, Two-way ANOVA. *n* = 8 sessions × 4 time points per group. Main effects: Learning versus Retrieval, *F*_(1,124)_ = 11.66, *p* = 0.0009; Goal-seeking versus Random-foraging, *F*_(1,124)_ = 0.0578, *p* = 0.8105; Interaction *F*_(1,124)_ = 0.6253, *p* = 0.4307. *Post hoc* multiple comparisons (Tukey's HSD): Learning-Goal versus Retrieval-Goal, adjusted *p* = 0.0130; Learning-Random versus Retrieval-Random, adjusted *p* = 0.0404; all other comparisons, adjusted *p* > 0.1399. ***F***, Wilcoxon rank sum test. *n* = 8 sessions × 4 time points per group. *p* = 0.2192. ***H***, Two-way ANOVA. *n* = 8 sessions × 4 time points per group. Main effects: Learning versus Retrieval, *F*_(1,124)_ = 1.582, *p* = 0.2111; Goal-seeking versus Random-foraging, *F*_(1,124)_ = 0.001244, *p* = 0.9719; Interaction, *F*_(1,124)_ = 0.007203, *p* = 0.9325. ***J***, Wilcoxon rank sum test. *n* = 8 sessions × 4 time points per group. *p* = 0.004707. ***L***, Two-way ANOVA. *n* = 8 sessions × 4 time points per group. Main effects: Learning versus Retrieval, *F*_(1,124)_ = 11.71, *p* = 0.0009; Goal-seeking versus Random-foraging, *F*_(1,124)_ = 0.3030, *p* = 0.5831; Interaction, *F*_(1,124)_ = 0.07663, *p* = 0.7824. *Post hoc* multiple comparisons (Tukey's HSD): Learning-Goal versus Retrieval-Goal, adjusted *p* = 0.0371; Learning-Random versus Retrieval-Random, adjusted *p* = 0.0457; Learning-Random versus Retrieval-Goal, adjusted *p* = 0.0386; all other comparisons, adjusted *p* > 0.1549. *p ≤ 0.05. **p ≤ 0.01. n.s., *p* > 0.05.

Given the observed changes in excitatory neural activity across experience, it was hypothesized that spatial representation at the ensemble level may change between the Learning and Retrieval phases. During movement epochs, the ongoing 6-12 Hz theta oscillation organizes hippocampal ensembles (Skaggs et al., 1996). Theta power increased throughout the Learning phase and plateaued during the Retrieval phase (Fig. 4*A–C*), parallel to the overall increase in excitatory firing rate (Fig. 3). Per-trial changes in theta power increased during both goal-seeking and random-foraging (Fig. 4*D*); however, changes in theta power were only correlated to goal-seeking, but not random-foraging, performance (theta power vs Goal latency, *r* = −0.154, *p* < 0.0160; vs Goal path length, *r* = −0.224, *p* < 0.000405; vs Random latency, *r* = −0.0841, *p* > 0.18; vs Random path length, *r* = 0.113, *p* > 0.077). The percent of recorded neurons that participated in each theta oscillation did not increase across learning (Fig. 4*E–H*); thus, the increase in population firing rate (Fig. 3) was likely driven by increased activity of individual neurons rather than recruitment of additional ensembles. Consistent with this hypothesis, the number of action potentials observed in individual participating neurons on each theta oscillation increased throughout the Learning phase and plateaued in the Retrieval phase (Fig. 4*I–L*). Due primarily to large variability in the activity of individual neurons on Trial 1, the specific windows used to define the Learning and Retrieval phases were impactful for participating cell activity analysis: a significant difference between Learning and Retrieval firing on an individual cell level was only observed when the Learning phase was at least three trials long and the Retrieval phase did not start until Trial 7 or 8 (Fig. 4*K*).

**Figure 5.**
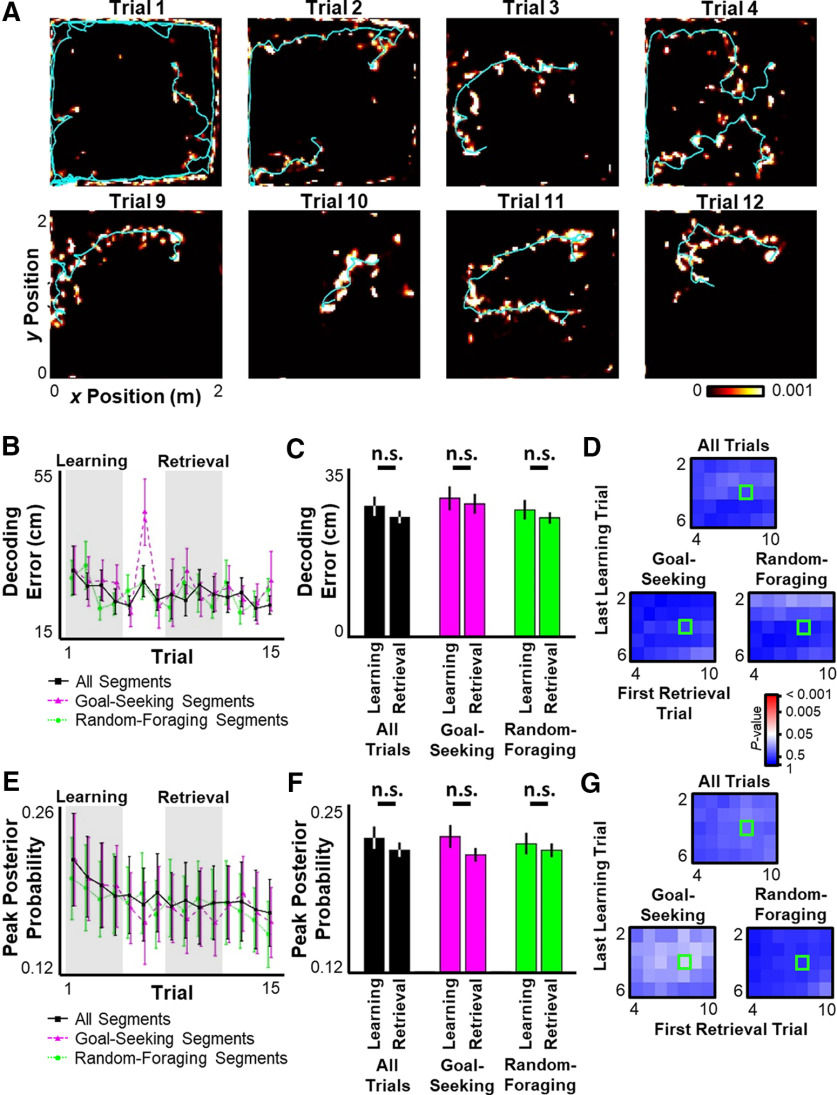
Spatial encoding across entire theta oscillations is unchanged by learning. ***A***, Ensemble activity during movement was decoded using a single decoding window for each theta oscillation. For representative trials across a single session, the mean decoded posterior probability across all theta oscillations within that trial (colormap) and the rat's behavioral trajectory (cyan line). For clarity, only the goal-directed portion of each trial is displayed. ***B***, ***E***, For the first 15 trials across all sessions, mean ± SEM decoding error (***B***) and maximal posterior probability (***E***) for each theta oscillation per trial. ***C***, ***F***, Mean ± SEM of decoding error (***C***) and maximal posterior probability (***F***) during Learning versus Retrieval periods. Black represents all theta oscillations during active movement. Magenta and green represent theta oscillations during either the goal-directed navigation (magenta) or random-foraging (green) component of the task. ***D***, ***G***, Significance matrix as in Figure 1*D* for decoding error (***D***) or maximal posterior probability (***G***). Statistical tests and results: ***C***, Two-way ANOVA. *n* = 8 sessions × 4 time points per group. Main effects: Learning versus Retrieval *F*_(2,186)_ = 1.326, *p* = 0.2510; Trial type *F*_(1,186)_ = 0.5138, *p* = 0.5991; Interaction *F*_(1,186)_ = 0.00847, *p* = 0.9916. ***F***, Two-way ANOVA. *n* = 8 sessions × 4 time points per group. Main effects: Learning versus Retrieval, *F*_(2,186)_ = 2.726, *p* = 0.1004; Trial type, *F*_(1,186)_ = 0.03736, *p* = 0.9633; Interaction, *F*_(1,186)_ = 0.2129, *p* = 0.8084. n.s., *p* > 0.05.

To assess how well the hippocampal ensemble encoded spatial information across time, a memory-less Bayesian decoding algorithm was applied to the ensemble activity across the entirety of each theta oscillation and the error between the decoded location and the rat's actual location (a measure of the accuracy of spatial representation) and the maximum posterior probability (a measure of the precision of spatial representation) were quantified (Fig. 5*A*). Neither network accuracy (Fig. 5*B–D*) nor precision (Fig. 5*E–G*) was significantly different across Learning and Retrieval phases within theta oscillations during active movement. These observations remained true when analyses were restricted only to goal-directed or random-foraging segments (Fig. 5*B–G*). Despite a lack of significant difference between the Learning and Retrieval phases, there is an apparent trend toward reduced decoding error and posterior probability across trials, particularly during the Learning phase (Fig. 5*B*,*E*). However, no statistically significant correlation was observed between either of these measures and trial number, perhaps because of large variability in decoding quality across sessions driven by differences in total cell yield (decoding error vs trial number, first 15 trials, *r* = −0.107, *p* > 0.24; first four trials, *r* = −0.027, *p* > 0.79; peak posterior probability vs trial number, first 15 trials, *r* = −0.113, *p* > 0.26; first four trials, *r* = −0.079, *p* > 0.44). Thus, while overall activity rates increased across learning, spatial encoding at the behavioral timescale did not appear to meaningfully change during active navigation.

**Figure 6.**
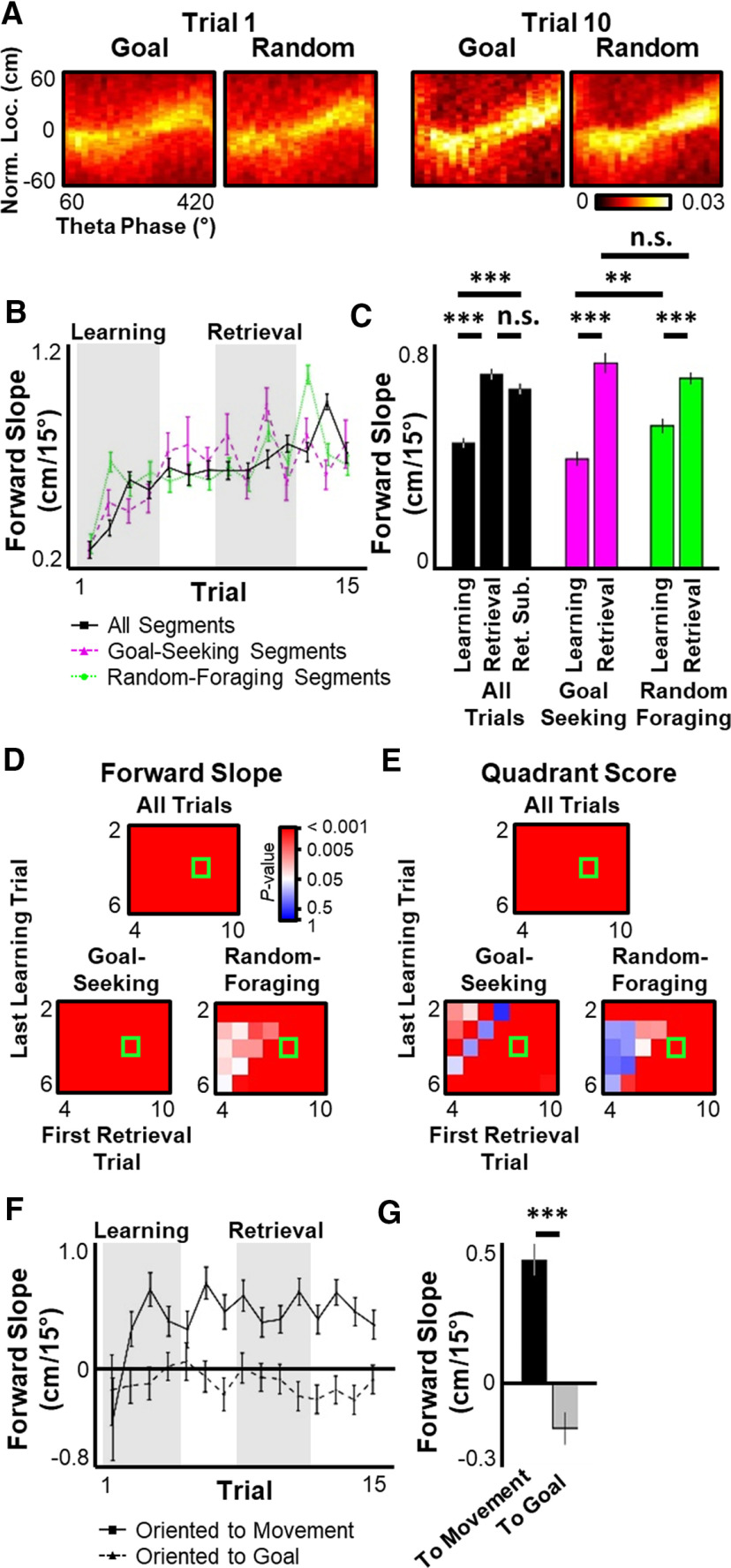
Theta sequences extend to movement direction, but not Goal location across learning. ***A***, Each theta oscillation during movement (velocity ≥ 10 cm/s) was decoded in 15° steps and normalized to rat's current position and movement direction. Plotted is the probability histogram of theta phase with maximal posterior probability at each position relative to rat position (2 cm bins) for all movement-related theta oscillations during goal-seeking (Goal) or random-foraging (Random) components of Trials 1 and 10. ***B***, For the first 15 trials across all sessions, mean ± SEM slope of weighted best-fit line of forward portion of decoded theta sequences oriented to rat's movement direction. ***C***, Mean ± SEM of forward slope during Learning versus Retrieval periods. Black represents all theta oscillations during active movement, including Retrieval period with subsampled spike data (Ret. Sub.). Magenta and green represent theta oscillations during either the goal-directed navigation (magenta) or random-foraging (green) component of the task. ***D***, ***E***, Significance matrix as in Figure 1*D* for forward slope of weighted best fit line (***D***) or quadrant score (***E***). ***F***, Same as in ***B***, for theta sequences when rat was within 25 cm of Goal well and the difference between the rat's movement direction and the direction to the Goal well was >30° and <180°. Each theta oscillation was oriented to either the rat's current movement direction (solid line) or to the direction to the Goal (dashed line). ***G***, Mean ± SEM of forward slope during Retrieval phase for theta sequences oriented to rat's movement direction (black) versus the direction to the goal well (gray). Statistical tests and results: ***C***, Left, One-way ANOVA. *n* = 13,551 Learning oscillations, 10,313 Retrieval and Retrieval Subsample oscillations. *F*_(2, 34,174)_ = 51.109; *p* < 10^−10^). *Post hoc* multiple comparisons (Tukey's HSD): Learning versus Retrieval, adjusted *p* < 10^−9^; Learning versus Ret. Sub., adjusted *p* < 10^−9^; Retrieval versus Ret. Sub., adjusted *p* > 0.13. ***C***, Right, Two-way ANOVA. *n* = 6892 Learning-Goal oscillations, 2953 Retrieval-Goal oscillations, 6659 Learning-Random oscillations, 7752 Retrieval-Random oscillations. Main effects: Learning versus Retrieval, *F*_(1, 24,252)_ = 82.56, *p* < 0.0001; Goal-seeking versus Random-foraging, *F*_(1, 24,252)_ = 1.243, *p* = 0.2649; Interaction, *F*_(1, 24,252)_ = 9.421, *p* = 0.0021. *Post hoc* multiple comparisons (Tukey's HSD): Retrieval-Goal versus Retrieval-Random, adjusted *p* = 0.5961; Learning-Goal versus Learning-Random, adjusted *p* = 0.0043; all other comparisons, adjusted *p* < 0.0001. ***G***, Wilcoxon rank sum test. *n* = 1162 oscillations. *p* < 10^−10^. **p ≤ 0.01. ***p ≤ 0.001. n.s., *p* > 0.05.

On a finer timescale, theta oscillations express brief virtual sweeps through the current environment, termed theta sequences (Skaggs et al., 1996; Foster and Wilson, 2007; Johnson and Redish, 2007; Gupta et al., 2012; Zheng et al., 2016; Kay et al., 2020; Wang et al., 2020). The quality of theta sequences has been reported to increase following learning (Feng et al., 2015; Igata et al., 2021), and theta sequences have been shown to encode goal locations during a task requiring precise spatial memory (Wikenheiser and Redish, 2015). To explore whether representation of the newly learned Goal location emerged in theta sequences across learning, each theta oscillation was decoded and examined at a fine timescale. The slope (i.e., the virtual movement velocity) of theta sequences increased throughout the Learning period and plateaued in the Retrieval phase (Fig. 6*A–D*), indicating that the hippocampal network became increasingly capable of prospectively evaluating more distant future paths as a function of experience. This learning-related increase in theta sequence quality was observed during both goal-seeking and random-foraging phases of the task (Fig. 6*C*,*D*). Unexpectedly, theta sequence slopes were significantly higher while the rat was engaged in random foraging behavior compared with goal-seeking in the Learning phase, although both behaviors displayed similar theta sequence quality in the Retrieval phase (Fig. 6*C*). As with excitatory firing rate and theta power, the improvement in theta sequence slope directly correlated with improved navigation to the Goal well, but not the Random well (forward slope vs Goal latency, *r* = −0.71, *p* < 0.0028; vs Goal path length, *r* = −0.70, *p* < 0.0038; vs Random latency, *r* = −0.21, *p* > 0.47; vs Random path length, *r* = 0.16, *p* > 0.58). To determine whether the increase in theta sequence quality was a direct result of increased overall firing rates in each theta oscillation (Fig. 4), the number of spikes in each theta oscillation for Trials 8-11 were randomly subsampled to match the average number observed in Trials 1-4 for that session. This subsampling was repeated 1000 times. The slope of the forward portion of the theta sequence was not significantly changed when the number of action potentials was matched between the Learning and Retrieval phases (Fig. 6*C*), arguing that the improvement in theta sequence quality was unlikely to be exclusively driven by increased network activity. The quadrant score of each oscillation (Feng et al., 2015), an additional measure of theta sequence quality, also improved across the Learning to Retrieval phases (Fig. 6*E*). As with the theta sequence slope, the quadrant score was significantly improved from Learning to Retrieval phases, even when the Retrieval phase was subsampled to match the number of action potentials observed in the Learning phase (one-way ANOVA, *F*_(2, 34,178)_, main effect *p* < 10^−6^, Tukey's HSD *post hoc* multiple comparisons Learning vs Retrieval and Learning vs Retrieval Subsampled adjusted *p* < 10^−6^).

To explore whether theta sequences preferentially encoded a path to the newly learned Goal location, each theta sequence was oriented to either the rat's current movement direction or the direction to the Goal well. Given the relatively short paths encoded by theta sequences (mean ± SEM, 7.83 ± 0.03 cm across all theta oscillations oriented to movement), analysis was restricted to times when the rat was within 25 cm of the Goal well. To dissociate theta sequence alignment to movement versus alignment to the goal, analysis was further restricted to periods when the difference between the rat's movement direction and the direction from the rat to the Goal well was <180° (the rat was not moving away from the Goal well) and >30° (the rat was not moving directly to the Goal well). In this subset, theta sequences were significantly more aligned with the rat's movement direction than with the direction to the Goal well, even after learning (Fig. 6*F*,*G*), indicating that, in this task, theta sequences did not facilitate goal-directed navigation by preferentially encoding paths to the Goal well.

**Figure 7.**
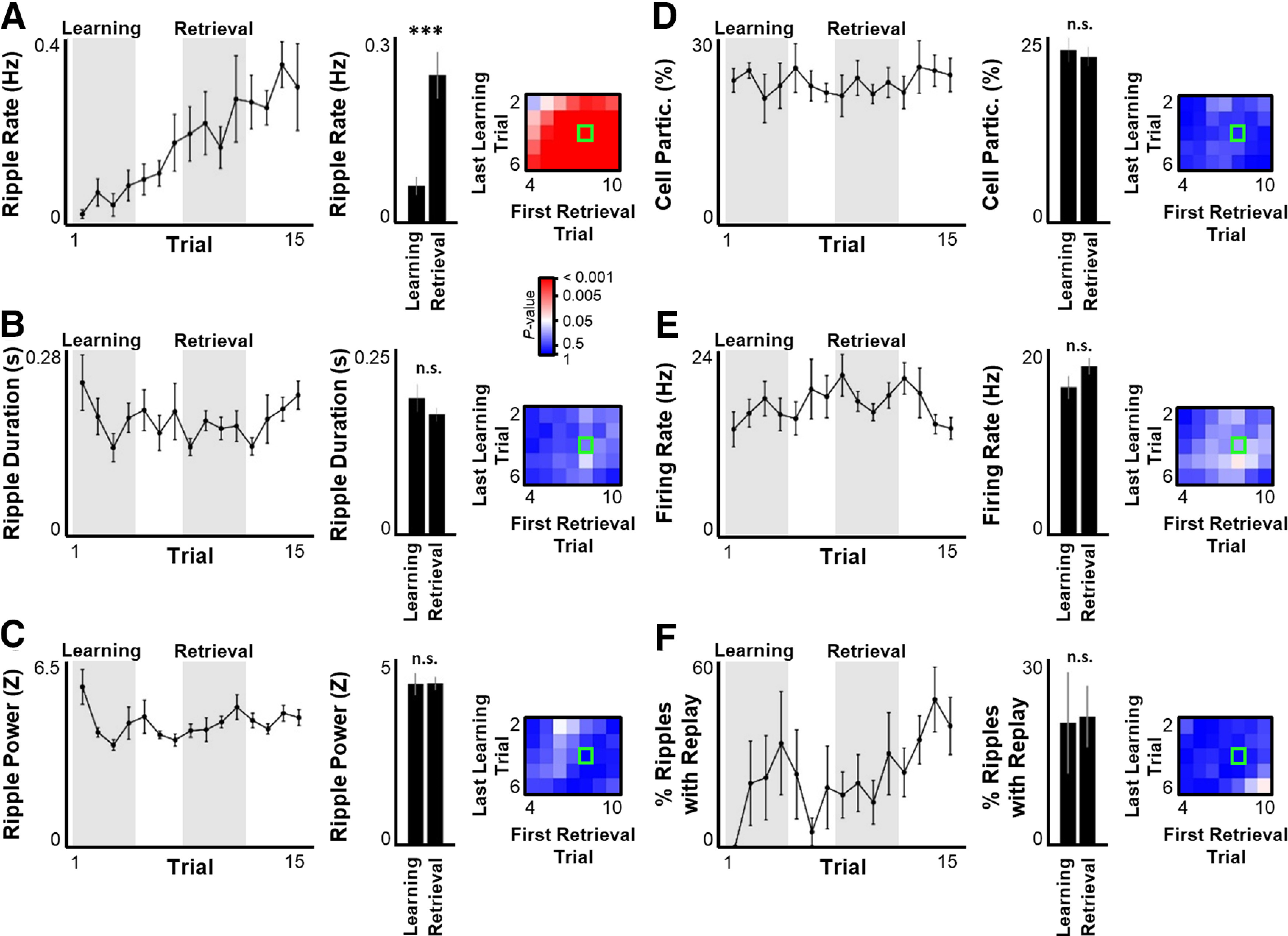
Ripple rate, but not other ripple properties, increases across learning. ***A-F***, Same as in Figure 4*A-C*, for ripple rate per time immobile (***A***), ripple duration (***B***), ripple power (***C***), percent of recorded cells that participate in each ripple (***D***), firing rate of participating cells per ripple (***E***), and percent of ripples that encode statistically significant replay (***F***). Statistical tests and results: ***A***, Wilcoxon rank sum test. *n* = 8 sessions × 4 time points per group. *p* = 8.495 × 10^−6^. ***B***, Wilcoxon rank sum test. *n* = 8 sessions × 4 time points per group. *p* = 0.5836. ***C***, Wilcoxon rank sum test. *n* = 8 sessions × 4 time points per group. *p* = 0.3647. ***D***, Wilcoxon rank sum test. *n* = 8 sessions × 4 time points per group. *p* = 0.5134. ***E***, Wilcoxon rank sum test. *n* = 8 sessions × 4 time points per group. *p* = 0.1458. ***F***, Wilcoxon rank sum test. *n* = 8 sessions × 4 time points per group. *p* = 0.3964. ***p ≤ 0.001; n.s., *p* > 0.05.

The increase in excitatory firing rates across learning (Fig. 3) may reflect increased input from upstream regions or may instead reflect increased excitability of the local hippocampal network. To explore these two possibilities, ensemble activity was examined within sharp-wave/ripple events, when hippocampal activity is thought to be largely driven by local, intrahippocampal sources (Buzsáki, 2015; Davoudi and Foster, 2019). The occurrence rate of ripples (number of ripples per time immobile) dramatically increased across learning (Fig. 7*A*), consistent with prior reports (Jackson et al., 2006; Eschenko et al., 2008; Girardeau et al., 2014; Igata et al., 2021) and a role for extrahippocampal input to drive ripple initiation (Abadchi et al., 2020). However, neither ripple duration, ripple power, cell participation, within-ripple firing rate, nor the percent of ripples that encoded statistically significant virtual spatial trajectories (replay events) was impacted by experience (Fig. 7*B–F*). These results indicate that, while ripple drive increased across learning, basic circuit properties underlying ripple expression were unchanged. Thus, it is likely that local hippocampal excitability is unaffected by experience in this task and the movement-based changes in excitatory firing rate (Fig. 3) and theta power (Fig. 4) are a result of increased extrahippocampal drive.

**Figure 8.**
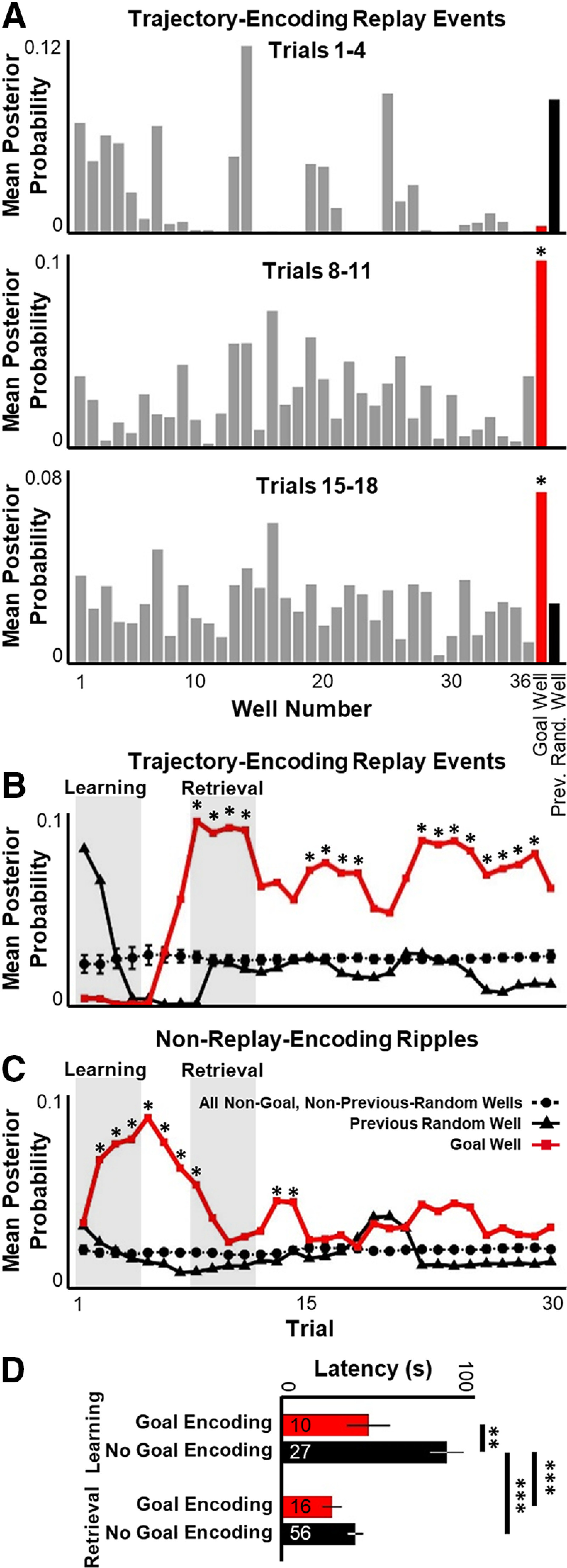
Representation of a goal emerges rapidly in ripples. ***A***, Across all sessions, mean representation of the Goal well (red), the previous Random well (black), and all other wells (gray) in all statistically significant replay events occurring in Trials 1-4 (top), Trials 8-11 (middle), and Trials 15-18 (bottom). For each replay, representation of the well currently occupied by the rat was eliminated from analysis. *p < 0.05 (statistical outlier, Grubb's test). ***B***, Representation of the Goal well (red), previous Random well (solid black), or average ± SEM of all other wells (dashed black) across the first 30 trials, using a four-trial average. *p < 0.05, Goal well is a statistical outlier from all other wells for that four-trial window (Grubb's test). The previous Random well is not a statistical outlier for any data point. ***C***, Same as in ***B*** for ripples that do not encode statistically significant, spatially smooth trajectories. ***D***, For both Learning (top) and Retrieval (bottom) phases, mean latency to return to Goal well following ripples which occurred while the rat was away from the Goal well and which represented the Goal well (“Goal Encoding,” red) or did not represent the Goal well (“No Goal Encoding,” black). Statistical tests and results: ***D***, Two-way ANOVA. *n* = 10 Learning/Goal-encoding ripples, 16 Retrieval/Goal-encoding ripples, 27 Learning/non-Goal-encoding ripples, 56 Retrieval/non-Goal-encoding ripples. Main effects: Learning versus Retrieval, *F*_(1,105)_ = 17.53, *p* < 0.0001; Goal-encoding versus non-Goal-encoding, *F*_(1,105)_ = 11.12, *p* = 0.0012; Interaction, *F*_(1,105)_ = 3.266, *p* = 0.0736. *Post hoc* multiple comparisons (Tukey's HSD): Learning/Goal-encoding versus Learning/non-Goal-encoding, adjusted *p* = 0.0086; Retrieval/Goal-encoding versus Learning/not-Goal-encoding, adjusted *p* < 0.0001; Learning/non-Goal-encoding versus Retrieval/non-Goal-encoding, adjusted *p* < 0.0001; all other comparisons, adjusted *p* > 0.5. *p ≤ 0.05.

The patterns of neuronal activity within hippocampal ripples often represent virtual spatial paths through the current environment (Diba and Buzsáki, 2007; Davidson et al., 2009; Pfeiffer and Foster, 2013, 2015; Pfeiffer, 2020). The spatial representation encoded within ripple events was quantified by identifying a subset of ripple events that encoded a statistically significant spatial trajectory (termed replay events). Prior work has demonstrated that replay events in this task are significantly biased to encode a path from the rat's current location to the recently learned Goal well (Pfeiffer and Foster, 2013). However, those analyses were averaged across the entire session and did not assess potential trial-to-trial changes in replay content as the rat learns the Goal well location. A hypothesis of the current study is that learning produces a rapid increase in the number of replay events that encode rat-to-Goal virtual trajectories, and thus, an increase in Goal representation that parallels improvements in behavioral performance. Alternatively, recent work suggesting that replay events preferentially encode prior reward locations predicts that emergence of the Goal in replay may not appear until considerably later in training (Carey et al., 2019; Gillespie et al., 2021). To test these alternate hypotheses, each replay event was analyzed to quantify the representation of the Goal well compared with the previous Random well and all other wells. Because of small numbers of ripple events in each trial, a running average representation across four consecutive trials was used. Importantly, for each replay event, the representation of the well currently occupied by the rat was eliminated to remove the well-known initiation bias of replay. This prevented artificially increasing the representation of the Goal well because of increased numbers of replay events that occurred while the rat was at that location, and thus explicitly focused the analysis on nonlocal representation in replay. Surprisingly, virtually no representation of the Goal well was observed in replay events during the Learning phase. Starting on Trials 7 and 8 (in the beginning of the Retrieval phase), a sudden increase in Goal representation arose in replay events which persisted throughout the remainder of the experiment (Fig. 8*A*,*B*). Thus, Goal representation in replay events only emerged after the rat had achieved peak performance on the task, suggesting that such events are unlikely to be necessary for initial learning. Representation of the previous Random well was observed strongly in replays of the first few trials, but this representation dropped rapidly during learning and encoding of the previous Random well was never significantly different from any other non-Goal well throughout the experiment (Fig. 8*A*,*B*), strongly indicating that, in this behavioral task, retrospective replay of nonrepeated past experience is rare. Prior work (Pfeiffer and Foster, 2013) demonstrated that the virtual trajectories encoded within replay events are moderately correlated with the rat's heading direction. To determine whether the increased representation of the Goal well was driven by replay events that occurred while the rat was facing the Goal well, replay events were subdivided into those in which the rat was oriented toward the Goal well (±45°) or was facing elsewhere. No significant effect of heading direction was observed (two-way ANOVA *F*_(1,24)_, all main effects *p* > 0.095).

Contrary to replay-encoding ripples, non–replay-encoding ripples showed a rapid, but transient, increase in Goal representation during the Learning phase (Fig. 8*C*). The criteria used to define replay events require representation of a spatially plausible path, meaning that ripples encoding trajectories which jump or teleport around the arena would fail to qualify. As with replay events, representation of the well currently occupied by the rat was eliminated from analysis of non–replay-encoding ripples. Thus, this analysis excluded ripple events which encoded stationary representation of the rat's current location (Denovellis et al., 2021). Goal representation in non–replay-encoding ripples therefore necessarily included a virtual teleportation of spatial representation to the Goal without a spatially informative virtual path leading to that location. This nonlocal Goal representation across all ripples arose very quickly in the Learning phase in a manner that preceded changes in movement-based network activity. Furthermore, the emergence of Goal representation within ripples on the first few trials strongly argues that the novel Goal location can be incorporated into hippocampal ripples without prolonged, repeated experience. As observed for trajectory-encoding replays, the Goal representation in non–replay-encoding ripples was not impacted by whether the rat was facing the Goal well at the time of the event (two-way ANOVA *F*_(1,24)_, all main effects *p* > 0.30).

To determine whether representation of the Goal well in ripples impacted subsequent behavioral performance, the latency of the rat to return to the Goal well was quantified for every ripple event that occurred while the rat was away from the Goal well. This analysis combined both trajectory-encoding replay events and non–replay-encoding ripples together. Significantly shorter latencies to return to the Goal well were observed following Goal-encoding ripples than following ripples which did not represent the Goal well (Fig. 8*D*). *Post hoc* analysis revealed that this effect was only significant for the Learning period (Fig. 8*D*), suggesting that representation of the Goal, even during nonspatially structured ripples, contributes to improved behavioral performance.

## Discussion

This work provides a detailed examination of hippocampal network modulation during a period of rapid spatial memory formation and subsequent memory retrieval. The study used a modified delayed matching-to-place task, a behavior shown to require proper hippocampal function and plasticity across both hippocampal and frontal cortical networks (Steele and Morris, 1999; Kilonzo et al., 2021). This work reveals four main findings: (1) the Goal location did not display elevated firing rates or accumulation of place fields, even after behavioral performance indicated that spatial memory of the Goal location had been established; (2) global activity rates increased across the hippocampal network during periods of active movement in a manner that paralleled behavioral performance; (3) the quality of theta sequences improved across learning without reliably encoding virtual trajectories to the learned Goal; and (4) the novel Goal location was rapidly encoded in spatially unstructured ripples which emerged during early learning periods, followed by a prolonged representation of the Goal in spatially organized replay during post-learning retrieval periods. Together, these findings provide evidence that successful spatial navigation does not require enhanced representation of a distant goal location across the hippocampal network during active movement. Rather, goal representation appears to initially emerge during periods of immobility, when hippocampal networks may be able to synchronize activity of cortical networks implicated in goal representation (Basu et al., 2021; Hocker et al., 2021).

The absence of amplified goal-specific encoding in place field activity is surprising given several prior studies that reported increased hippocampal representation of a learned site of reward (Hollup et al., 2001; Lee et al., 2006; Hok et al., 2007; Dupret et al., 2010; Mamad et al., 2017; Kaufman et al., 2020). Most prior work exploring goal encoding used tasks that maintained the goal in a consistent spatial location across sessions (Hollup et al., 2001; Lee et al., 2006; Hok et al., 2007; Mamad et al., 2017; Kaufman et al., 2020). In the present study, however, the Goal location moved unpredictably between sessions, which may account for the lack of accumulation of goal-related activity reported here. Importantly, two previous studies observed increased goal representation in place cell activity even when the goal location changed daily (Dupret et al., 2010; Xu et al., 2019). In these two reports, rats displayed navigation “errors” during the initial trials of each day, exploring the previous day's rewarding sites before learning the current day's goal (Dupret et al., 2010; Xu et al., 2019). In contrast, rats in the present study did not visit the previous day's Goal location more often than chance (across-session binomial cumulative distribution *p* > 0.68; in only one session did a rat explore the previous day's Goal well before finding the current Goal well on Trial 1). This may be because of prolonged training in the current study, during which rats adopted an optimal strategy of avoiding Goal sites from previous days, as they were never reused on subsequent days. Furthermore, most prior work on goal representation in the hippocampus has relied on environments with linear paths or open environments with consistent optimal path strategies, such that the rat necessarily follows the same path and reactivates the same population of neurons on each goal-directed trajectory. In the current study, however, rats were forced to navigate to a goal location in an open field from a large number of starting locations; thus, paths to the Goal well rarely repeated. This lack of repetition may prevent or slow synaptic plasticity which likely underlies accumulation of goal representation across the hippocampal network (Dupret et al., 2010). Finally, it is possible that increased Goal representation may require prolonged experience, and may have emerged in the present study had the experiment been extended. Regardless, these data indicate that successful spatial memory retrieval is not critically dependent on accumulation of place fields at a learned goal location.

In opposition to the lack of change in goal representation across learning, several features of hippocampal function during active movement did change in parallel to mnemonic improvement. Excitatory firing rate, theta power, and the quality of theta sequences all significantly increased as rats learned the location of a goal and remained elevated during sustained performance. These changes directly correlated to improved navigation toward the learned Goal well, but did not relate to random foraging success, indicating a specific effect on memory-guided behavior (rather than, for example, heightened sensitivity to olfactory cues). Importantly, these changes in online network activity did not arise exclusively at the learned Goal location or emerge only during periods of goal-directed navigation but were instead observed throughout the task and across the entire population of recorded place cells. These findings argue that, for this spatial navigation task, increased place cell representation exclusively at a learned reward location is not necessary for improved goal-directed navigation to that site. Rather, global activity of excitatory hippocampal neurons increased with learning, consistent with a prior study (Michon et al., 2021). Such increases in overall firing rates are unlikely to be due to increased speed (McNaughton et al., 1983), as velocity was unchanged across learning. The fact that firing rates increased regardless of place field location and during both the random foraging and goal-directed navigation portions of the task strongly suggests a global increase in synaptic input as a function of experience. Furthermore, measures of local connectivity and excitability during hippocampal ripples were unchanged across learning. Given that activity in ripples is largely thought to be driven by local, intrahippocampal connections (Buzsáki, 2015; Davoudi and Foster, 2019), the increase in “online” activity without a concomitant increase in “offline” activity is consistent with the hypothesis that extrahippocampal input may be the driving force behind the changes in online activity observed in the hippocampal network, although more work is necessary to conclusively test this hypothesis. Prior reports indicate that hippocampal theta power is increased during periods of spatial decision-making (Schmidt et al., 2013; Belchior et al., 2014), supporting a model of hippocampal function in which increased network organization during active navigation facilitates information retrieval and/or processing.

Surprisingly, however, the increase in theta power and improvement in the quality of theta sequences did not appear to result in reliable encoding of theta sequences to the learned Goal location. A prior report demonstrated that theta sequences can encode paths to distant goal locations in a circular track (Wikenheiser and Redish, 2015). This prior study further indicated that theta sequences were generally correlated to behavioral needs, with longer sequences emerging during navigation to more distant goals (Wikenheiser and Redish, 2015). A subsequent study, however, reported that the spatial content in theta sequences does not predict future behavior, but instead iteratively samples between multiple possible future paths (Kay et al., 2020). In the current study, goal representation in theta sequences was assessed by orienting the decoded virtual paths to either the rat's movement direction or the direction to the Goal. In this task, theta sequences are heavily biased to reflect the rat's current movement direction, even when that movement was not optimally oriented to the learned Goal. Given the lack of representation of the Goal location in theta sequences, even when the rat was near the Goal, it is unclear whether or how theta sequences might facilitate navigation to the goal in the present task.

During periods of immobility, the hippocampus produces brief, highly organized activity bursts termed ripples, which often encode spatially structured virtual trajectories through the current environment (Foster and Wilson, 2006; Diba and Buzsáki, 2007; Gupta et al., 2010; Pfeiffer and Foster, 2013, 2015; Pfeiffer, 2020). In this study, nonlocal goal representation emerged rapidly in ripples which did not appear to encode spatially coherent replay of virtual paths. Representation of the goal in non–replay-encoding ripples diminished after learning, and was replaced by goal representation in ripples which encoded coherent replay. Thus, across all ripples, representation of the Goal location emerged rapidly and persisted throughout the entirety of the experiment. While many diverse paths were encoded by ripples in this dataset (Pfeiffer and Foster, 2013), only the Goal well showed repeated, elevated representation, a finding observed on virtually every trial. The previous Random well was not encoded in ripples more strongly than any other non-Goal well, suggesting that, in this task, replay did not preferentially encode paths to prior rewarding locations (Gillespie et al., 2021). Further, the rapid emergence of Goal representation in ripples in the current study argues against a slow, progressive development in which past rewarding sites are gradually incorporated into ripples (Gillespie et al., 2021). Importantly, this work does not refute that of prior studies which showed a lack of current goal representation in ripples (Carey et al., 2019; Gillespie et al., 2021), but rather argues for a more nuanced view of hippocampal ripples as a flexible mechanism that may be capable of altering their information content based on the cognitive demands of the current task. The transition from unstructured ripples to spatially organized replay across the first few trials argues that experience-dependent synaptic plasticity, perhaps across the CA3 or CA2 networks (He et al., 2021), is necessary to coordinate replay, even in a familiar environment.

Recent work indicates that neurons in the orbitofrontal cortex can faithfully encode spatial information regarding distant goal locations (Basu et al., 2021; Hocker et al., 2021). Goal representation in the orbitofrontal cortex emerges before movement onset and is maintained throughout the entirety of goal-directed navigation (Basu et al., 2021). Hippocampal activity in ripples is known to activate and coordinate activity across a large number of cortical networks (Logothetis et al., 2012; Shin et al., 2019; Abadchi et al., 2020). Thus, findings from the current study support a model of hippocampo-cortical function in which hippocampal ripples can represent a distant goal location before movement, thereby activating orbitofrontal cortex neurons that also encode that location and which serve to maintain representation of that goal during subsequent navigation.
